# Acute Zonal Occult Outer Retinopathy (AZOOR): a case report of vision improvement after intravitreal injection of Ozurdex

**DOI:** 10.1186/s12886-017-0638-5

**Published:** 2017-12-06

**Authors:** Yi Chun Kuo, Nancy Chen, Rong Kung Tsai

**Affiliations:** 1 Department of Ophthalmology, Buddhist Tzu Chi General Hospital, Hualien, Taiwan; 2 Institute of Eye Research, Buddhist Tzu Chi General Hospital, Tzu Chi University, 707, Sec. 3, Chung-Yang Rd., Hualien, 970 Taiwan; 3 Institute of Medical Sciences, Tzu Chi University, Hualien, Taiwan

**Keywords:** Acute zonal occult outer retinopathy, Optical coherence tomography, Electroretinogram, Sustained-released steroid

## Abstract

**Background:**

AZOOR was first described by Gass in 1993 as a syndrome with rapid loss of one or more extensive zones of the outer retinal segments. It is characterized by photopsia, minimal funduscopic changes, and electroretinographic abnormalities. The efficacy of systemic steroids in treating AZOOR has been previously described and advocated by the concept of autoimmune retinopathy. However, the use of intravitreal of sustained-released steroid had not been mentioned to date.

**Case presentation:**

A 34-year-old man had sudden onset of central scotoma and photopsia in the left eye. His visual acuity continued deteriorating. The visual field defect demonstrated bilateral enlarged blind spots and altitudinal defects. Fluorescein angiography (FA) showed nonspecific retinal inflammation, and an electroretinogram (ERG) illustrated decreased amplitude of the b wave in both eyes. Optical coherence tomography (OCT) examinations revealed parafoveal loss of the photoreceptor inner/outer segment (IS/OS) junction. Therefore, acute zonal occult outer retinopathy (AZOOR) was diagnosed. Although his vision did not improve under the initial treatment of systemic corticosteroid and calcium channel blocker, remarkable improvement was noticed after the intravitreal injection(IVI) of Ozurdex, consistent with the recovered IS/OS junction disruption.

**Conclusions:**

We herein report a typical case of AZOOR, suggesting that the intravitreal injection of steroid may benefit in certain patients.

## Background

AZOOR was first described by Gass in 1993 as a syndrome with rapid loss of one or more extensive zones of the outer retinal segments. It occurs predominantly in young women, characterized by photopsia, minimal funduscopic changes, and electroretinographic abnormalities affecting one or both eyes [1, 2]. The most common visual field (VF) defects are enlarged blind spots and scotoma connecting to blind spots [3]. Retinal pigment epithelial (RPE) atrophy and retinal vessel narrowing have been observed in many cases as sequelae [4]. Optical coherence tomography (OCT) examinations demonstrated loss and irregularity of the IS/OS boundary in scotomatous areas [5, 6]. Fundus autofluorescence may also reveal a markedly hyperautofluorescent delineating line around the zonal area of RPE atrophy [7, 8]. The cone outer segment tip (COST) line has been recognized as a thin, highly reflective line located between the IS/OS junction and the RPE, and abnormalities of the COST line are observed in AZOOR [9, 10]. The efficacy of systemic steroids in treating AZOOR has been previously described. Visual improvement was achieved in most reported cases [11, 12]. Herein, we present a patient whose symptoms did not subside with oral steroid use; however, he experienced visual improvement after receiving intravitreal injection of a sustained-released steroid.

## Case presentation

The 34-year-old man was in good health until he failed to hit a ping-pong ball accurately one morning. Blackish dots appeared in the nasal upper visual field, and flashes blocked the vision in front of his left eye. He was myopic with −11.0 diopter (D) in the right eye (OD) and −15.0 in the left eye (OS), and he underwent LASIK surgery seven years ago. He visited our emergency department, where visual acuity (VA) revealed 20/20 (OD) and 20/32 (OS). No cells were noted at the anterior chamber (AC) or vitreous. His fundus seemed normal under dilated fundoscopy.

**Fig. 1:**
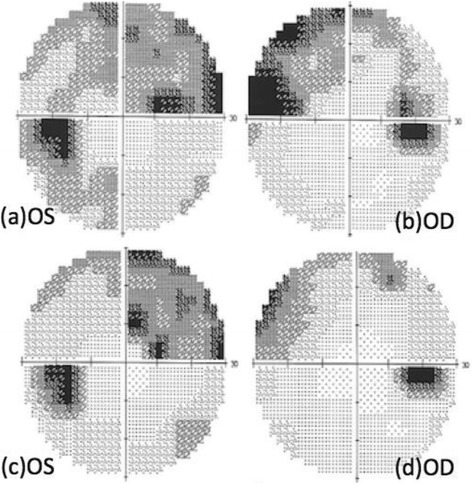
VF defect (detected by Humphrey Visual Field Analyser 30–2) significantly subsided after one month of IVI of Ozurdex. (**a**, **b**) VF on the next day after onset of symptoms. (OD: Fixation losses: 1/21, false positive errors: 5% and false negative errors: 6%. OS: Fixation losses: 0/21, false positive errors: 0% and false negative errors: 5%) (**c**, **d**) One month after IVI, VF defect significantly subsided of both eyes. (OD: Fixation losses: 0/19, false positive errors: 4% and false negative errors: 7%. OS: Fixation losses: 1/21, false positive errors: 0% and false negative errors: 1%)

**Fig. 2:**
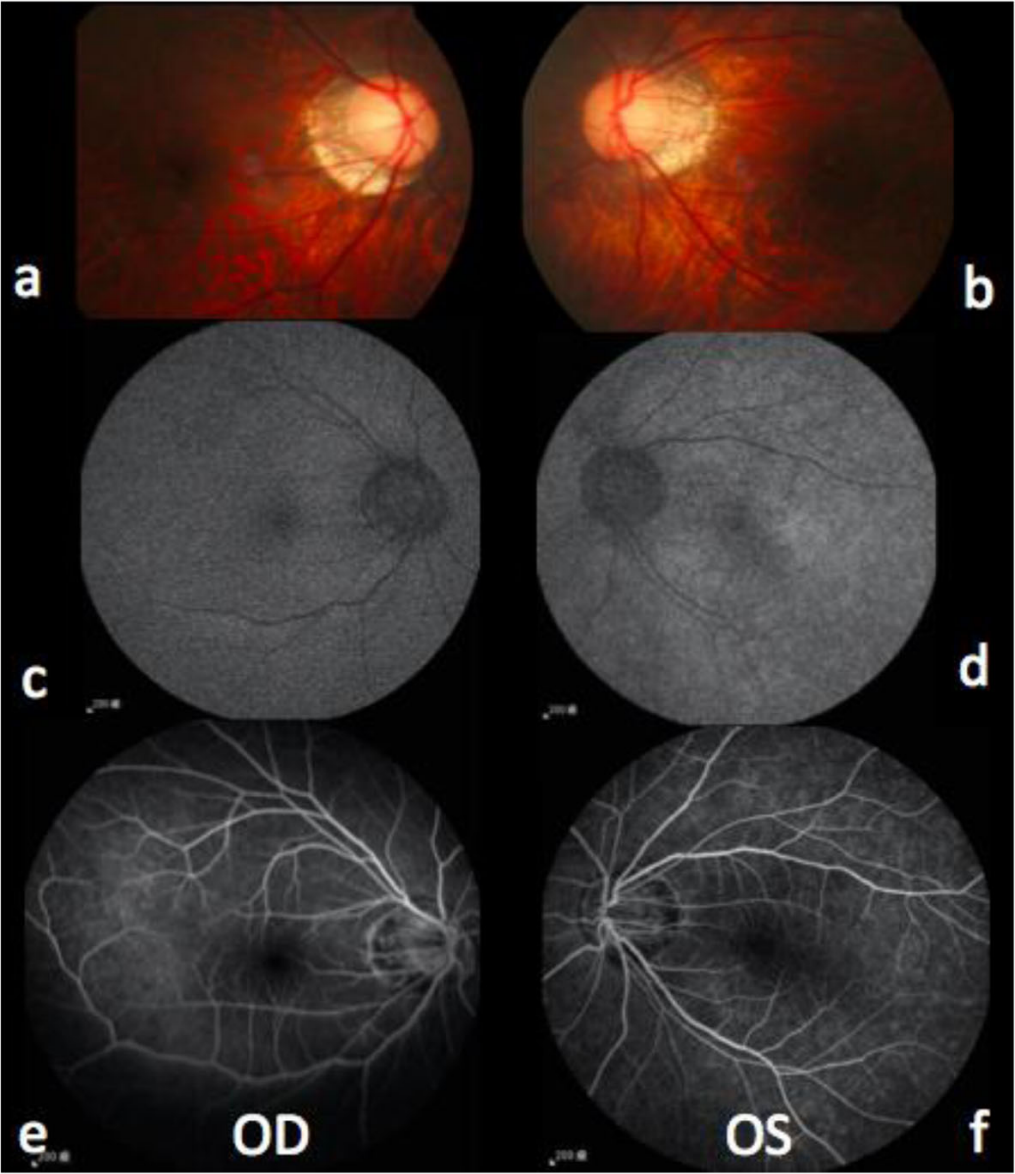
Binocular images of color fundoscopy, fundus autofluorescence, and fluorescein angiography on the next day after onset of symptoms. Color fundoscopy (**a**-**b**) revealed no specific finding except myopic crescent. Compared to the right eye (**c**), fundus autofluorescence revealed hyper-autofluorescence of the left eye (**d**). Fluorescein angiography (**e**-**f**) revealed retina small vessels vasculitis inflammation of the left eye(**f**)

**Fig. 3:**
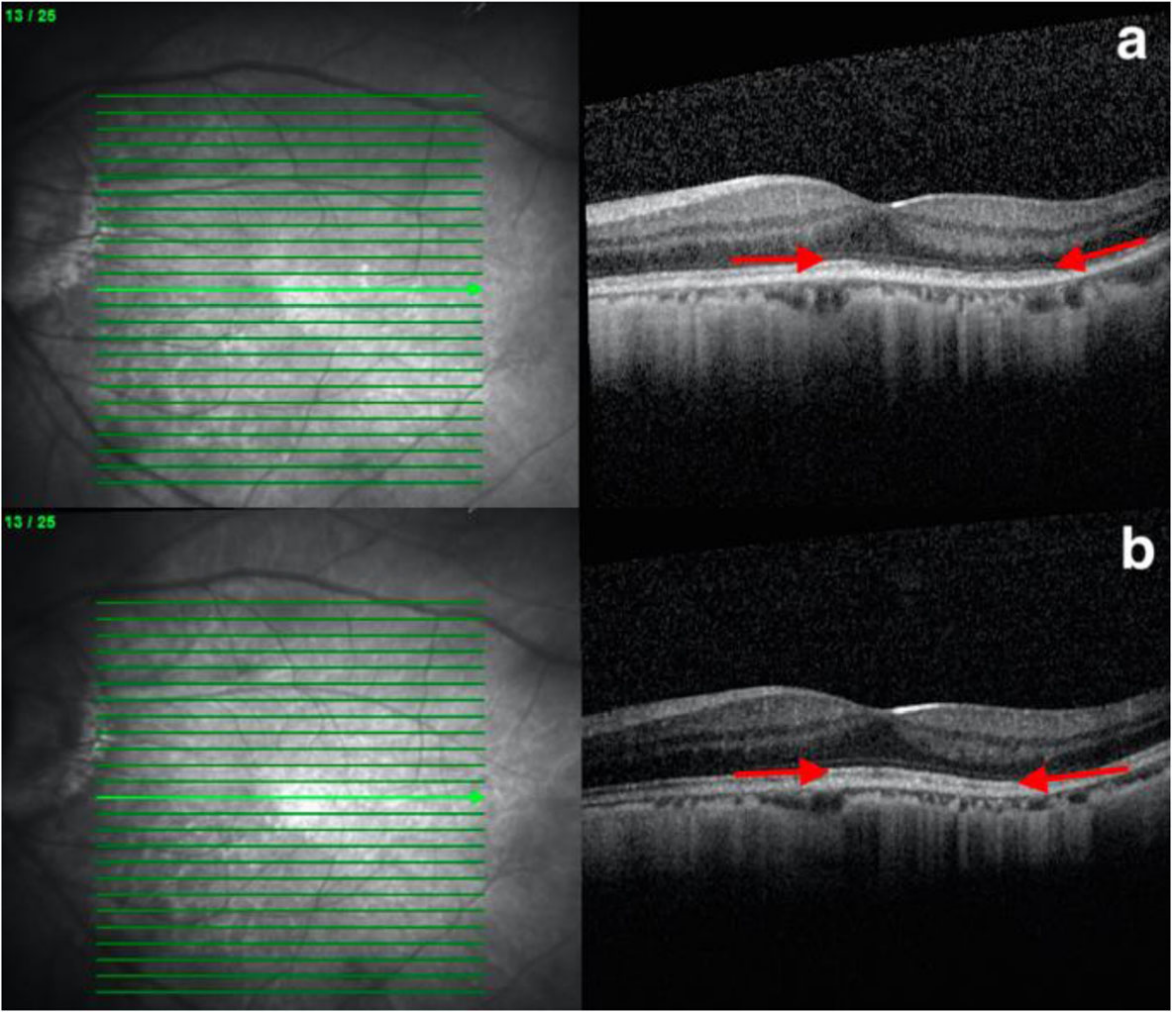
OCT revealed change of IS/OS junction before and after treatment. **a** On the next day after onset of symptom, OCT revealed parafoveal loss (between arrows) of the photoreceptor IS/OS junction. **b** OCT revealed restoration (between arrows) of IS/OS junction after two weeks of IVI of Ozurdex

**Fig. 4:**
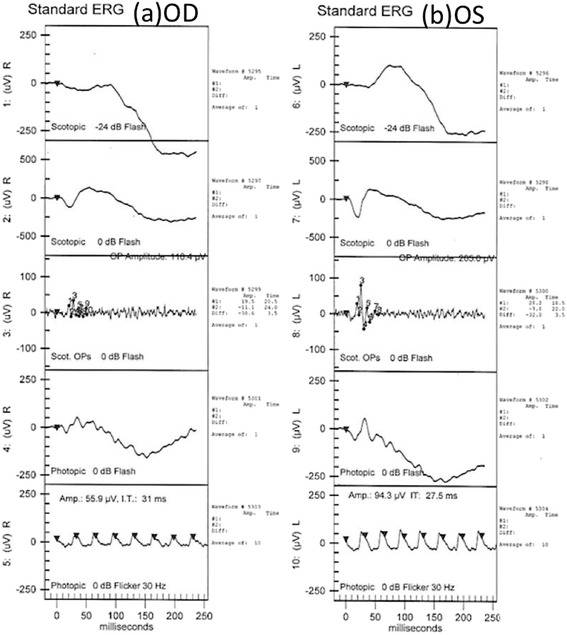
(**a**, **b**) ERG illustrated decreased amplitude of b wave of scotopic stimulations in both eyes

On the next day, vision decreased significantly to 20/25 (OD) and 20/63 (OS). Visual field (VF) examinations demonstrated bilateral enlarged blind spots and altitudinal defects (Fig. 1a-b). FA showed hyperfluorescence (OS) and inflammation of retinal small vessels (Fig. 2). OCT examinations revealed parafoveal loss of the photoreceptor IS/OS junction (Fig. 3a). ERG revealed decreased amplitude of the b wave in both eyes (Fig. 4a-b). According to his symptoms and examinations, AZOOR was diagnosed. We gave systemic corticosteroid (dexamethasone 6 mg once a day) on the next day after day of onset. Nevertheless, his vision didn’t improve after dexamethasone treatment for 9 days, so we tapered dexamethasone to 4 mg and prescribed calcium channel blocker (amlodipine 5 mg once a day). However, his VA did not improve for 3 weeks. Intravitreal injections (IVI) of Ozurdex (OS) was suggested and arranged. The next day after IVI of Ozurdex, his VA improved to 20/32 (OS). The course was smooth. Follow-up OCT showed improvement of the IS/OS junction disruption two weeks later (Fig. 3b). VA (OS) improved to 20/20, and the VF defect improved significantly (Fig.1c-d) as of one month after IVI. Owing to the appearance of the high myopia tilting disc and deep vertical enlarged cupping, the VF defects might be a summation of different components, AZOOR and myopia related glaucoma. Intraocular pressure (IOP) was elevated to 28 mmHg initially after IVI; however, IOP remained within normal range after 2 weeks of bimatoprost (Lumigan) treatment. At the recent follow-up after 13 months of the episode, his best corrected VA (OS) was 20/20. There was no cataract nor IOP elevation; meanwhile, OCT showed no sign of IS/OS junction disruption (Table 1).

**Table 1:** Relevant medical history and interventions

Date	Summaries from Initial and Follow-up Visits	Diagnostic Testing	Interventions
2015/11/20	vision deteriorated(OS)	visual acuity (VA)20/20 (OD) and 20/32 (OS)	
2015/11/24	Still central scotoma (OS)	FA: hyperfluorescence (OS) and inflammation of retinal small vessels. OCT: parafoveal loss of the photoreceptor IS/OS junction	Dexamethasone 6 mg daily
2015/12/02	Still central scotoma (OS)	stationary	Taper dexamethasone to 4 mg daily, and add Amlodipine 5 mg daily
2015/12/15	Still central scotoma (OS)	stationary	Intravitreal injection of Ozurdex.
2015/12/30	Vision improved (OS)	VA (OS) improved to 20/20 OCT: improvement of the IS/OS junction disruption	
2017/01/18	Vision stable(OS)	VA (OS) was 20/20 OCT: no sign of IS/OS junction disruption.	

## Discussion and conclusions

The etiology of AZOOR remains controversial. An infectious viral process of the outer retina was suggested by Gass. [1] Autoimmune and inflammatory hypotheses were proposed by Jampol and Becker [13, 14]. Other possible mechanisms includes fungal infiltration, [15, 16] polycythemia vera, [17] toxic retinopathy, [14, 18, 19] and anti-retinal antibody [20, 21]. Heckenlively and Ferreyra [22] reviewed retinal disorders evoked by autoantibodies and established a clinical term of autoimmune retinopathy (AIR). They stated that AIR could be a secondary complication of AZOOR, implying that an autoimmune reaction mediated by humoral immunity plays an important role in the disease process of AZOOR. Logically, steroid therapy might be effective in some AZOOR patients [11, 23].

Various treatments have been attempted in patients with AZOOR, including systemic corticosteroids, [11, 12, 24] other systemic immunosuppressive agents, [4, 25] and antimicrobial agents, [19] but their effects were not conclusive yet. Based on the proposed mechanisms of anti-retinal antibody, [20–22] AZOOR could be an autoimmune disease with anti-retinal antibody leakage from the disc margin with spread of immune substance under the retina. [21] Previous studies have showed effectiveness of systemic steroid. Saito [24] gave fourteen patients intravenous methylprednisolone 1000 mg/day for 3 days, oral prednisolone 30 mg/day for 7 days, intravenous methylprednisolone 1000 mg/day for 3 days, with prednisolone monthly tapering with 30 mg/day, 20 mg/day, 15 mg/day, 10 mg/day, 5 mg/day (each dose for one month). Their final logMAR BCVA (0.16 ± 0.52) was significantly better than the pre-treatment value (0.56 ± 0.60) (*P* = 0.007), and the mean MD at the final visit (−9.82 ± 10.74 dB) was significantly higher than the initial value (−13.66 ± 10.73 dB) (*P* = 0.02). Chen [12] gave three patients intravenous pulse steroid therapy (methylprednisolone 1000 mg/day for 3 days) as the initial treatment followed by oral prednisolone with gradual tapering within 3 months, and prescribed another five patients oral prednisolone 1 mg/kg/day as the initial treatment with gradual tapering in 3 months. Although all of the patients had improvement of visual field eventually; however, recurrence was noted in two patients and three patients experienced deterioration of the visual field after stopping or tapering the systemic steroid. Immunotherapy with azathioprine [24] or mycophenolate [12] was added to patients with reactivation of the disease during prednisolone taper.

On the other hand, ocular penetration of systemically administered drugs could have been restricted by the blood–retinal barrier, and the diluting effect of blood volume requires larger systemic doses [26]. Ozurdex contained micronized dexamethasone 0.7 mg in a biodegradable copolymer, and could be effective up to 6 months after intravitreal injection [26]. Hence, IVI of steroid might be beneficial for patients with AZOOR in the rationale.

Studies have demonstrated that visual functions in AZOOR patients improved spontaneously, [24] whereas systemic steroid treatment showed benefits to visual outcome as well [12, 24]. Meanwhile, improvement of visual field defects was correlated with choroidal thickness reduction, [27] possibly implying “inflammatory” pattern in the choroid in the acute stage [28]. Our AZOOR case is the first to show improved VA and recovery of the IS/OS junction after IVI of Ozurdex. During the 13-month follow-up, his condition remained stable. However, we cannot rule out the residual or delayed effects from the systemic steroid treatment received prior to the administration of Ozurdex or the possibility of spontaneous recovery. Therefore, the reliability of intravitreal injection treatment with sustained released steroid need further elucidation with future clinical research of AZOOR.

## Abbreviations

AIR: Autoimmune retinopathy
AZOOR: Acute zonal occult outer retinopathy
COST: The cone outer segment tip
D: Diopter
ERG: Electroretinogram
FA: Fluorescein Angiography
IOP: Intraocular pressure
IS/OS junction: Inner/outer segment
IVI: Intravitreal injection
OCT: Optical coherence tomography
RPE: Retinal pigment epithelial
VA: Visual acuity
VF: Visual field
